# Application of temporary patient-specific plate in head and neck osteocutaneous free flap reconstruction: a prospective pilot study

**DOI:** 10.3389/froh.2026.1692401

**Published:** 2026-02-20

**Authors:** Yui Yin Ko, Yu-xiong Su, Jingya Jane Pu

**Affiliations:** Division of Oral and Maxillofacial Surgery, The University of Hong Kong, Sai Ying Pun, Hong Kong SAR, China

**Keywords:** 3D printing, computer-assisted surgery, free flap, jaw reconstruction, virtual surgical planning

## Abstract

**Background:**

During autologous osteocutaneous free flap transfer, the traditional process of manually contouring harvested bone segments to match the virtually planned jaw morphology is often imprecise and time-consuming. Consequently, the use of virtual surgical planning (VSP) and patient-specific surgical plates (PSPs) has gained substantial popularity in overcoming such challenges. However, the high costs and complex equipment required for manufacturing PSPs may render this technology inaccessible.

**Purpose:**

This study aimed to investigate the feasibility of developing a resin-made temporary patient-specific plate (TPSP) as an adjunct to improve the surgical efficiency and accuracy of reconstructive surgeries.

**Study design, setting, and sample:**

A prospective, single-centre study was performed. Patients indicated for computer-assisted free osteocutaneous flap reconstruction were recruited between December 2020 and October 2021.

**Outcome variables:**

Patient demographics, intraoperative characteristics, and postoperative outcomes were reviewed. Reconstruction accuracy was evaluated by comparing preoperative plans and postoperative models; the deviations and angulations of the reconstructed jaw, bone segments, and dental implants were measured.

**Analyses:**

Descriptive analysis was carried out.

**Results:**

Ten patients (seven men, three women) with a mean age of 52.9 ± 18.8 years were included in the study. In terms of surgical efficiency, the mean operative time was 492 ± 84.2 min, while the mean plating and reconstructive times were 58.7 ± 19.4 and 131.7 ± 26.4 min, respectively. A total of 26 simultaneous dental implants were placed in eight patients. One (3.8%) implant was abandoned due to intra-operative fenestration of the fibula bone, and one implant was explanted 12 months postoperatively. In terms of accuracy, the mean absolute distance deviation was 0.621 ± 0.548 (range: 0.0047–1.6277). The mean distance and deviation of the reconstructed segments were 5.16 ± 2.25 mm and 9.67° ± 6.84°, respectively. The mean distance deviations at the implant platform and apex were 3.94 ± 2.20 and 3.95 ± 2.97 mm, respectively, with a mean implant deviation of 12.8° ± 9.94°.

**Conclusions and relevance:**

The use of TPSP proved to be a more affordable intraoperative alternative to PSP for aligning and stabilizing bony segments during flap inset while achieving surgical efficiency and accuracy comparable to those of PSP.

## Introduction

Microsurgical osteocutaneous flap transfer is frequently employed to restore bony continuity following extensive maxillofacial ablative surgery. Depending on the size and complexity of the composite defect, commonly harvested osteocutaneous free flaps for the maxillofacial region include the fibula free flap, deep circumflex iliac flap, and scapular free flap. Traditionally, jaw reconstruction is performed using free-hand segmental osteotomies at the donor site, followed by intraoperative recontouring and plating of the bony segments. Owing to the complex geometry of the maxillofacial region and its proximity to the digestive and respiratory tracts, a high degree of precision is required to restore facial form, speech, and mastication functions (1). Suboptimal reconstruction can result in facial deformity, malocclusion, and the need for complex subsequent occlusal rehabilitation. Therefore, virtual surgical planning (VSP) and three-dimensional (3D) printing technologies have emerged as promising fields to address these challenges.

The adoption of VSP technology offers multiple advantages. First, it allows for clear visualization of the extent of pathology in the maxillofacial region, enabling preplanned resection planes while accounting for vital structures (2). Furthermore, studies have shown that VSP flattens the learning curve for junior surgeons, leading to success rates in bony union for mandibular fibular free flap reconstructions comparable to achieved by senior surgeons with significantly more clinical experience (3). In addition, VSP can reduce operating room costs by shortening operative times by up to 60 min (4, 5).

Advancements in 3D printing technology have enabled the fabrication of patient-specific cutting guides, reconstructed jaw models, and patient-specific plates (PSPs), enhancing the visualization of surgical plans. The high degree of flexibility in plate and screw placement afforded by PSPs enables individualized customization, reducing the risk of hardware-related complications, including screw loosening, hardware extrusion, and the need for hardware removal (6). The adoption of VSP also allows for careful consideration in positioning donor bone segments to maximize bone apposition and osseous vascularity, thereby improving long-term bony union rates (7). Moreover, it increases intra-operative predictability in complex double-barrel fibula configurations. This technique enables simultaneous restoration of the vertical height required for dental implant-supported prosthetic rehabilitation while ensuring symmetrical restoration of the mandibular inferior border and chin contour (8).

PSPs, in particular, play a key role in facilitating the intra-operative execution of VSP. Their unique morphology and predesigned screw hole positions assist surgeons in precisely orienting bony segments to achieve the planned jaw shape. However, the high precision requirements of PSPs in the maxillofacial region necessitate the use of selective laser melting (SLM) or electron beam melting (EBM) technologies. Consequently, the high costs and complex equipment required for manufacturing these titanium implants may render such technology inaccessible in many regions (9). With the increasing availability of open-source surgical planning software and the growing affordability of 3D printers, resin-made patient-specific devices represent a more cost-effective alternative to PSP.

Thus, our centre developed a novel 3D-printed temporary patient-specific plate (TPSP). The TPSP closely resembles a patient-specific surgical plate and is made from ISO-certified, biocompatible, autoclavable PA2200 polyamide (Electro Optical Systems, Germany). This resin exhibits excellent strength and durability, with a high tensile modulus of 1,650 MPa and a flexural modulus of 1,500 MPa. In addition, it has a high melting temperature of 176°C and is available in fine powder form, enabling detailed 3D printing using selective laser sintering (10). These properties make PA2200 an ideal autoclavable and dimensionally stable material for the production of surgical guides. The TPSP facilitates the alignment and stabilization of bony segments, enabling the assembly of bony segments into the planned jaw configuration at the donor site and direct transfer to the ablative defect, thereby minimizing ischaemic and reconstruction time.

Hence, our pilot study aimed to investigate the feasibility of the TPSP device as an intraoperative adjunct to improve surgical efficiency and accuracy in reconstructive surgeries.

## Materials and methods

### Patient selection

A prospective single-centre study was conducted. Patients indicated for computer-assisted free osteocutaneous flap reconstruction were recruited between December 2020 and October 2021 at the Department of Oral and Maxillofacial Surgery, Queen Mary Hospital, Hong Kong. Patients with failed free flap reconstruction and intraoperative deviations from the virtual surgical plan were excluded. Ethical approval was obtained from the Institutional Review Board of The University of Hong Kong, Hospital Authority Hong Kong West Cluster (UW20-548), and written informed consent was obtained from all participants. This study was conducted in full accordance with the Declaration of Helsinki. All patients underwent a computed tomography (CT) scan 1-month postoperatively and were reviewed every month during the first postoperative year, every 2 months in the second year, every 3 months in the third year, and every 4–5 months during the fourth and fifth years.

### Virtual surgical planning and surgical workflow

The VSP workflow employed at our centre has been outlined in our previous publications (11–13). In brief, preoperative CT scans of the skull and CT angiography of the lower limbs were first performed in all cases. Preoperative CT imaging was acquired using a General Electric Revolution system (GE Healthcare, Chicago, IL, USA), with a slice thickness of 0.625 mm for both the head and neck and lower limbs. An iodinated contrast medium (Iopamiro 370, Bracco, Israel), with a concentration of 370 mg/mL, was administered intravenously at a flow rate of 4–5 mL/s. CT angiography of the lower extremity was performed during the arterial phase. The imaging data were exported in Digital Imaging and Communications in Medicine (DICOM) format.

Virtual surgical planning was then performed using ProPlan CMF 2.0 software (Materialise, Leuven, Belgium), followed by in-house design of 3D-printed surgical guides and TPSPs using 3-matic 13.0 software (Materialise). The design of the TPSP closely resembled the morphology of patient-specific surgical plates (PSPs), and likewise, the fixation hole positions were predesignated to match the drill holes created during osseous segment harvesting. This design facilitated manipulation of the osseous segments into the planned jaw morphology. The TPSPs were printed using ISO-certified, biocompatible, and autoclavable PA2200 polyamide (Electro Optical Systems, Germany), with a thickness of 3 mm.

**Figure 1:**
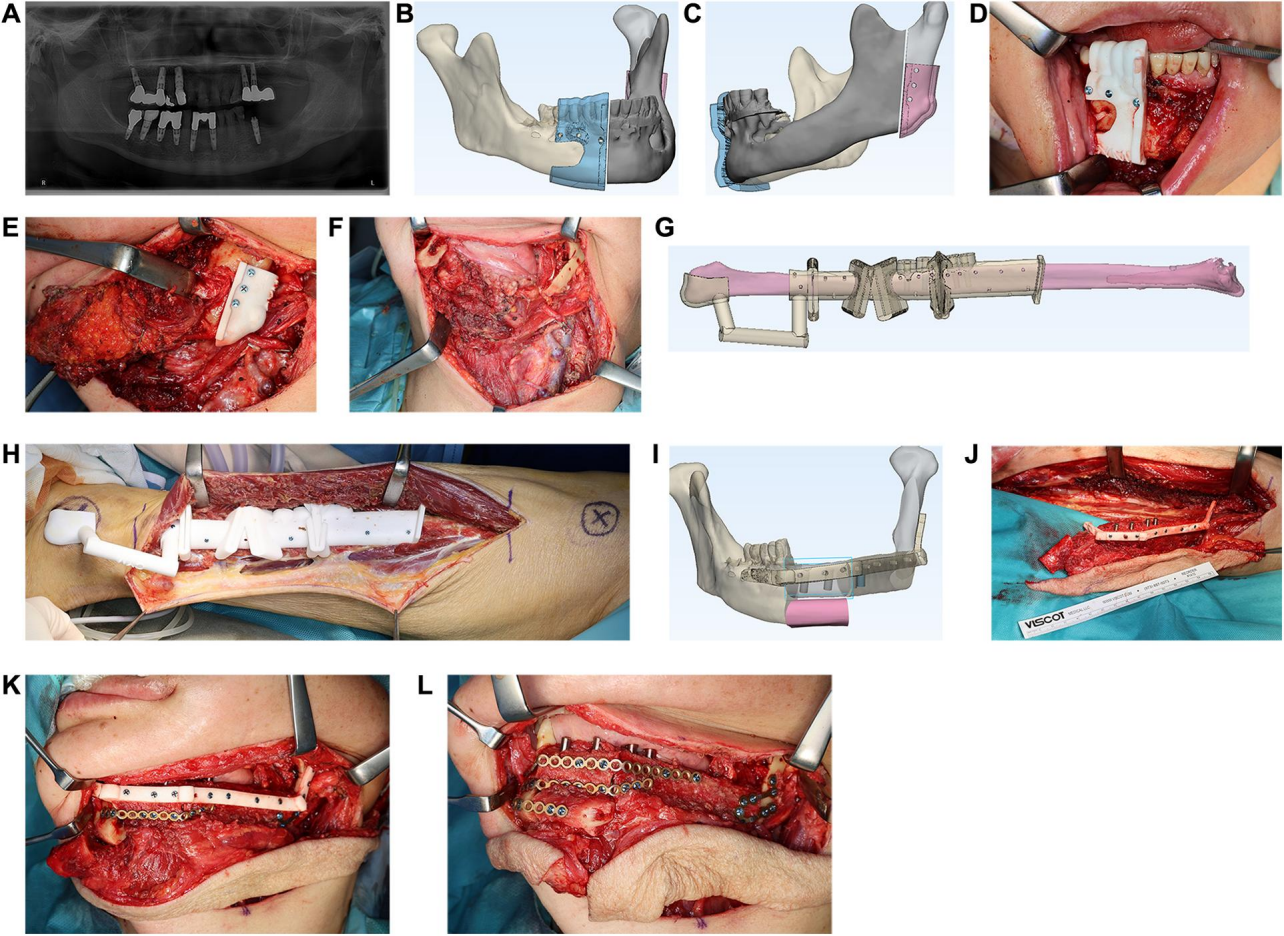
Mandibular reconstruction with fibula free flap after partial mandibulectomy for intraosseous squamous cell carcinoma. **(A)** Orthopantomogram demonstrating an osteolytic lesion at the left mandible involving the mandibular canal. **(B,C)** VSP of the mandibular resection guide. **(D,E)** Mandibular resection guide fitted intraoperatively with screws. **(F)** Mandibular defect following guided resection. **(G)** VSP of fibula segmentation guides with an in-built implant placement guide. **(H)** Fibula segmentation guide fitted intraoperatively with screws. **(I)** VSP of the double-barrel mandibular reconstruction and TPSP. **(J)** Assembly of the neomandible with TPSP at the donor site prior to dividing the pedicle. **(K)** Fibula free flap transferred and temporarily stabilized with TPSP to enable fixation of conventional miniplates. **(L)** Final fixation of fibula segments following removal of TPSP.

Three-dimensional-printed surgical guides were used during jaw resection, harvesting of the osseous free flap, and placement of simultaneous dental implants (Figures 1B–F). After segmentation of the osseous free flap, the bony segments were aligned and stabilized using the TPSP at the donor site, in accordance with the patient's 3D morphology and the predesignated matching screw holes, before dividing the pedicle (Figures 1G,H). During flap inset, vascular microanastomosis to the recipient vessels was performed, followed by temporary fixation of the bony flap to the remnant of the jaw using the TPSP (Figure 1L). After that, conventional plates were used for fixation, followed by the removal of the TPSP (Figure 1M).

### Outcome assessment

Patient demographics, intraoperative characteristics, and postoperative outcomes were prospectively collected. These included pathological diagnoses of lesions, defect locations, the need for postoperative adjunct therapy, and postoperative events. Intraoperative characteristics recorded included total operating time, plating and reconstruction times, intraoperative events, and length of postoperative hospital stay. The reconstruction time was defined as the interval from pedicle division to completion of neo-jaw fixation with conventional plates at the recipient site. The plating time was defined as the time from completion of vascular anastomosis to completion of neo-jaw fixation with conventional plates at the recipient site.

### Accuracy

**Figure 2:**
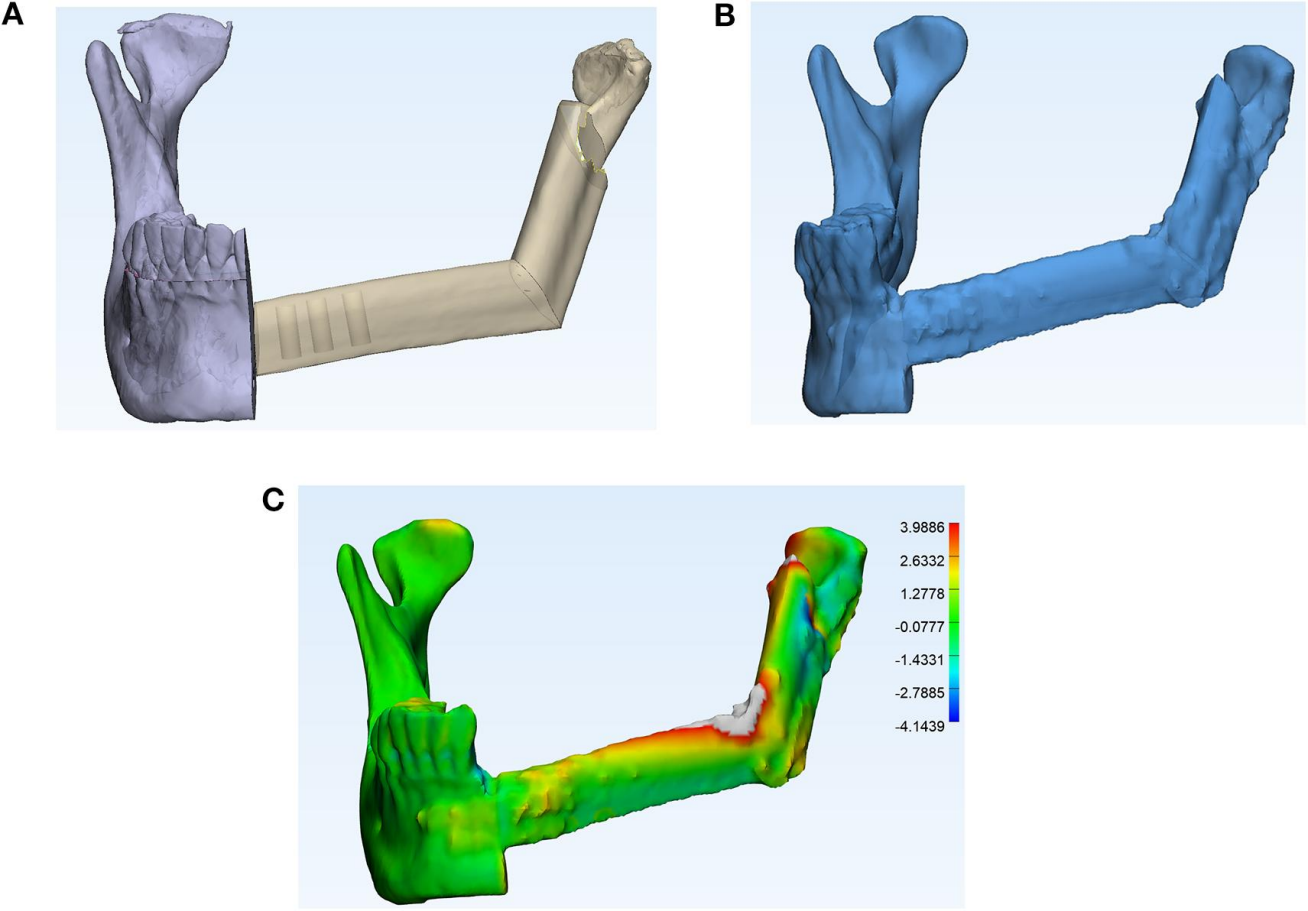
Accuracy analysis of the reconstruction: **(A)** 3D model of the planned mandible reconstruction with fibula free flap; **(B)** 3D model of the postoperative reconstructed jaw; and **(C)** superimposition (with heatmap) of the planned reconstruction against the postoperative mandibular reconstruction. This alignment was performed with the contralateral jaw, and a comparison was made using a Part Comparison Analysis in 3-matic 13.0 software (Materialise). The deviation of the postoperative fibula segments (in grey) from the virtual model (in rainbow gradient) is displayed. Green indicates no deviation, while the intensity of red and blue colours corresponds to greater degrees of deviation (in mm).

The methodologies for accuracy analysis have been outlined in detail in our previous publications (12, 14). Three-dimensional reconstructed jaw models were generated from CT scans using ProPlan CMF 2.0 software. The planned and immediate postoperative models were then superimposed onto the best-fit contralateral jaw using 3-matic 13.0 software. The absolute distance deviation between the preoperatively planned model and the postoperative model was calculated using the built-in part comparison analysis and visualized as a heatmap (Figure 2). For mandibular reconstruction cases, intercondylar and intergonial distances and their deviations were also compared. To investigate the accuracy of the reconstruction, the centre of gravity and longitudinal axis of each reconstructed bone segment were generated. Absolute distance and angular deviations were derived from the linear distance between the two centres of gravity and the longitudinal axis, respectively. To determine the accuracy of simultaneous dental implant placement, the longitudinal axes of the implants were compared with the planned positions to derive the angular deviation. The centre point of the implant platform and apex were identified as the intersection points of the longitudinal axis with the top and bottom of the dental implant. The distance deviations of the platform and apex were measured and subsequently compared.

### Statistical analysis

All data were analysed descriptively. Continuous data were analysed using the Mann–Whitney *U* test to compare the clinical variables of benign and malignant cases. Statistical analysis was performed using the IBM Statistical Package for the Social Sciences version 29.0.2.0 (IBM Corp., Armonk, NY, USA), with a significance level of 5% applied.

## Results

### Patient characteristics

Between December 2020 and October 2021, 10 consecutive patients (mean age: 52.9 years ±18.8) who underwent maxillary or mandibular reconstruction using osteocutaneous free flaps with the aid of a TPSP were included in this study. The majority of cases were operated using a submandibular approach (8/10), with the facial artery serving as the main recipient vessel in most cases (9/10). The postoperative follow-up rate was 100%, and four patients received postoperative radiotherapy. The mean (range) follow-up duration was 41.1 (35.6–47.0) months. The average length of postoperative hospital stay was 10.4  ± 2.91 days.

Patient demographics and reconstruction characteristics are summarized in Table 1.

**Table 1:** Clinical characteristics of the study group.

Age/Gender	Primary diagnosis	Clinical staging	Brown classification	Type of reconstruction	Number of simultaneous dental implants	Postoperative adjunctive therapy	Surgical approach	Anastomosis recipient vessel
Malignancy								
74/F	SCC	IV	Mandible II	FFF	4	RT	Submandibular	Facial artery
30/M	Epithelioid hemangioendothelioma	IV	Maxilla IIb	FFF	4	N/A	Modified Weber–Ferguson	Facial artery
80/M	SCC	IV	Mandible III	FFF	N/A	N/A	Lower lip split	Lingual Artery
39/M	Chondrosarcoma	II	Mandible IV	*FFF*	N/A	C-RT	Submandibular	Facial artery
58/M	Intraosseous SCC	IV	Mandible IV	FFF	3	RT	Submandibular	Facial artery
68/M	SCC	II	Maxilla IIb	FFF	2	RT	Submandibular	Facial artery
Benign								
35/M	Ameloblastoma	N/A	Mandible III	FFF	5	N/A	Submandibular	Facial artery
54/F	Ameloblastoma	N/A	Mandible II	DCIA	3	N/A	Submandibular	Facial artery
28/M	Ameloblastoma	N/A	Mandible II	FFF	3	N/A	Submandibular	Facial artery
63/F	Osteoradionecrosis	N/A	Mandible Ic	FFF	2	N/A	Submandibular	Facial artery

F, female; M, male; SCC, squamous cell carcinoma; FFF, fibular free flap; DCIA, deep circumflex iliac artery flap; RT, radiotherapy; C, chemotherapy.

### Clinical efficiency

In terms of surgical efficiency, the mean operative time was 492 ± 84.2 min, while the mean plating and reconstructive times were 58.7 ± 19.4 and 131.7 ± 26.4 min, respectively (Table 2). One (3.8%) implant was abandoned due to intraoperative fenestration of the fibula bone. Intraoperative breakage of the TPSP occurred in one patient; however, a spare TPSP was available and was used without complication. On average, 5.6 conventional plates were used per patient for jaw fixation. The intraoperative fit of the TPSP was excellent and did not require further modification, thereby facilitating the bending of all conventional plates during the procedure.

**Table 2:** Mean total operative time, ischaemic time, and plating and reconstructive times of the study group.

Mean operative time (min)	Total (*n* = 10)	Malignant (*n* = 6)	Benign (*n* = 4)	*p*-Value
Total operative time	492 ± 84.2	497 ± 109	470 ± 22.8	0.915
Ischaemic time	66.2 ± 14.2	64.9 ± 11.9	68.2 ± 18.8	0.915
Plating time	58.7 ± 19.4	54.5 ± 19.6	58.3 ± 21.9	0.748
Reconstructive time	131.7 ± 26.4	54.5 ± 19.6	128.2 ± 34.8	0.522

### Accuracy analysis

The mean absolute distance deviation was 0.621 ± 0.548 mm (range: 0.0047–1.6277 mm). Among the eight patients who underwent mandibular reconstruction, the mean intercondylar distance deviation was 3.24 ± 2.46 mm, with a corresponding angular deviation of 3.94° ± 2.34°; the mean intergonial distance deviation was 1.84 ± 0.618 mm, with a corresponding angular deviation of 4.83° ± 2.25°. A total of 26 reconstructed bone segments were analysed. The mean distance deviation and angular deviation of the reconstructed segments were 5.16 ± 2.25 mm and 9.67° ± 6.84°, respectively.

A total of 26 simultaneous dental implants were placed in eight patients. The mean distance between the planned and postoperative implant positions at the implant platform and apex were 3.94 ± 2.20 and 3.95 ± 2.97 mm, respectively, and the mean angular deviation of the implants was 12.8° ± 9.94°.

### Complications

One patient required repositioning of dental implants 2 months postoperatively; one implant was explanted 12 months postoperatively. One patient presented with implant thread exposure secondary to osteoradionecrosis. Four patients required subsequent plate removal secondary to plate exposure, three of whom had received postoperative radiotherapy. The average time to plate exposure was 17.5 months.

## Discussion

This manuscript presents our design of the TPSP as an intraoperative adjunct for osteocutaneous free flap reconstruction of the maxillofacial region. The traditional method of manually assembling fibula segments into the planned jaw shape is both time-consuming and prone to rotational errors. In contrast, the application of the TPSP provides guided positioning and alignment of harvested bone segments, facilitating their assembly into the planned jaw morphology and enhancing the precision of jaw reconstruction.

Our surgical guides and TPSPs are printed with ISO-certified, biocompatible, and autoclavable PA2200 polyamide (Electro Optical Systems, Germany). Three-dimensional resin printing is significantly more affordable than titanium printing for PSPs. The estimated cost of a PSP for a two-segment fibular mandibular reconstruction per patient is approximately £3,750.50 (∼$4,900 USD), with predicted in-house savings of 11%–20% (15). Moe et al. estimated that the total startup cost for 3D resin printing—including two commercial 3D printers and five reels of resin filament—was $434.64 USD, resulting in a mean material cost of $3.87 USD per case for printing patient-specific fibula/maxillofacial models (16). Therefore, our resin-made surgical guides and TPSPs not only represent a cost-effective alternative to PSP but also make in-house, surgeon-driven design and fabrication of patient-specific surgical templates considerably more achievable.

### Clinical efficiency and outcomes

The influence of the TPSP on clinical efficiency can be evaluated by comparison with a historical cohort from our centre reported by Yang et al. (12). In that study, patient-specific fibula osteotomy guides, resection guides, and 3D-printed patient-specific titanium plates were utilized. Our study demonstrated comparable surgical efficiency, with an average operative time of 473.5 min compared to 510 min in the historical cohort. This improvement in surgical efficiency may be attribute to the learning curve associated with increasing experience in computer-assisted surgeries at our centre (17). With regard to implant survival outcomes, our study achieved an osseointegration rate of 96%, with only one implant requiring explantation during the follow-up period. The success rate of our cohort is consistent with findings from a systematic review and meta-analysis by Panchal et al., which reported implant survival rates of 94.7% among 1,084 patients with vascularized bone flaps (18).

### Accuracy analysis

Despite the increasing popularity of VSP-guided osteocutaneous free flap reconstruction, objective analyses of reconstruction accuracy remain scarce in the medical literature. By superimposing postoperative-generated models onto planned models, the accuracy of reconstruction in our study was evaluated and compared with that of a historical cohort from our centre (12). Our study demonstrated similar accuracy, with a mean absolute distance deviation of 0.62 ± 0.55 mm, compared to 1.5 ± 0.5 mm in patients receiving patient-specific implants. Similarly, the intercondylar distance deviation in our cohort (3.24 ± 2.46 mm) was comparable to that observed in patients receiving patient-specific implants (2.6 ± 3.0 mm).

When comparing the accuracy of our reconstructed bone segments, our angular deviation (9.67° ± 6.84°) was higher than that documented by Schepers et al., who reported a mean angular deviation of 4.2° ± 3.2° (19). This discrepancy may be attributed to the relatively high proportion of complex defects in our cohort, with 60% requiring reconstruction with three or more bony segments. The accuracy of CAD-CAM mandibular reconstruction is known to decrease as the number of fibula segments increases (20).

In VSP, the shape and positioning of donor bone segments are designed to maximize bony contact; therefore, any deviations from the planned configuration may result in bony gaps, which could significantly impact the long-term biomechanical strength of the reconstructed jaw. Ideally, the reconstructed mandible should achieve a fracture threshold comparable to that of a native jaw, which can endure occlusal forces of up to 250 N (21). Segmental resection of the mandible has been shown to reduce bite force by 76% in the molar region and by 59% in the incisor region, underscoring the importance of considering biomechanical factors in jaw reconstruction. These considerations are vital for achieving secure and stable fixation, thereby restoring early masticatory function (21). Finite-element analysis conducted by Wan et al. demonstrated that gaps between donor bone segments and the native jaw hinder effective force transmission within the bone and to the titanium plates. This results in increased stress levels on the plates, elevating the risk of hardware fracture and bony malunion (22). To enhance surgical precision and alignment with VSP, the incorporation of titanium inserts in fibula resection guides may improve saw guidance and reduce the risk of creating false routes, resulting in better alignment and fewer bony gaps (23).

Regarding the accuracy of simultaneous dental implants in our cohort, our results were comparable to those reported for patients receiving PSPs, as reported by Schepers et al. (19), who found a mean centre-point distance deviation of 3.3 mm (± 1.3 mm) and a mean angular deviation of 13.0° (± 6°) in their dental implants. These findings indicate that our TPSP design can achieve accuracy levels comparable to PSPs when performing simultaneous dental implants. As the intraoperative fitting of the TPSP was satisfactory in our cohort, axial angulation errors in our dental implants may have resulted from individual fibula anatomical variations of the fibula and subtle compromises in the fit of the fibula cutting guides secondary to uneven periosteum thickness (24).

While the use of the TPSP demonstrated promising results with accuracy comparable to that of PSPs, the clinical significance of these findings should be interpreted with caution. To date, no studies have established the minimally acceptable clinical deviation for VSP-guided maxillofacial reconstruction; hence, the impact of deviations in reconstructed bony segments and dental implants on aesthetic outcomes and subsequent prosthesis delivery remains to be truly elucidated. Nevertheless, the quantitative data provided in this study may serve as reference values in future studies aimed at establishing the accuracy and minimally acceptable clinical deviation in VSP-guided maxillofacial reconstruction.

Given the high fabrication costs and possible prolonged turnover times associated with PSPs (2), several alternative strategies have been proposed by surgeons to facilitate accurate positioning. For instance, titanium plates can be prebent on the 3D-printed reconstructed jaw models (25). Another strategy involves a drill-hole sharing technique, whereby screw holes created during fibula osteotomy and resection are aligned with those in the reconstructive plate to confirm that the positions of the bony segments are as planned (26). The use of patient-specific fixation trays has also been advocated to assist with fibula segment assembly (26–28). However, the bulky design of fixation trays may limit their ability to assemble into double-barrel configurations. Our TPSP design offers several advantages, including direct visualization of the planned jaw shape during assembly and facilitation of direct transfer to the ablative site for concurrent fixation, thereby minimizing translational errors. The neo-jaw is also stabilized by the TPSP during flap inset, enabling safe intraoperative plating while minimizing the risk of trauma to the anastomosed vessels. Furthermore, the amount of resin required to print the TPSP is significantly less than that needed to print patient-specific resin jaw models, thereby reducing medical waste and minimizing its subsequent environmental impact.

The main limitations of our study include the small sample size and the absence of a control group. Comparing our data with other studies may be challenging due to bias arising from differences in reconstructive defects, measurement parameters, software platforms, surgical workflows, and surgeon experience. Another limitation is that this study focused solely on the measurement of the accuracy of hard tissue reconstruction. Since the aim of reconstruction is the restoration of facial form and aesthetics, future studies could incorporate photomorphometric analysis to evaluate the accuracy of postoperative soft tissue reconstruction. Nevertheless, when compared with our historical cohort, the results from our case series are encouraging, demonstrating TPSP as a cost-effective adjunct to improve surgical efficiency and accuracy.

Technological advances in VSP and 3D printing have expanded the frontiers of preoperative planning in maxillofacial reconstruction. Our novel 3D-printed TPSP serves as an alternative to PSPs for the alignment, stabilization, and inset of bony flap segments, yielding comparable results to those of PSPs in terms of surgical efficiency and accuracy while reducing the printing costs and turnover time. Future studies involving larger sample sizes will be beneficial in consolidating the role of TPSP as a cost-effective alternative to PSPs.

## Data Availability

The original contributions presented in the study are included in the article, further inquiries can be directed to the corresponding author.
